# Toxicity Studies on Novel *N*-Substituted Bicyclo-Heptan-2-Amines at NMDA Receptors

**DOI:** 10.3390/ph6040536

**Published:** 2013-04-12

**Authors:** Natalia Coleman, Zeynep Ates-Alagoz, Boyenoh Gaye, Michelle Farbaniec, Shengguo Sun, Adeboye Adejare

**Affiliations:** 1 Department of Biological Sciences, Misher College, University of the Sciences, Philadelphia, PA 19104, USA; E-Mails: n.colema@usciences.edu (N.C.); mfarbaniec@mail.usciences.edu (M.F.); 2 Department of Pharmaceutical Sciences, Philadelphia College of Pharmacy, University of the Sciences, Philadelphia, PA 19104, USA; E-Mails: z.alagoz@usciences.edu (Z.A.-A.); bgaye@mail.usciences.edu (B.G.); shengguo20031@yahoo.com (S.S.); 3 Department of Pharmaceutical Chemistry, Faculty of Pharmacy, Ankara University, Tandogan 06100, Ankara, Turkey

**Keywords:** NMDA receptor antagonist, neurodegeneration, cytotoxicity, MDCK and N2a cells

## Abstract

Several novel norcamphor derivatives were designed and synthesized as uncompetitive NMDA receptor antagonists at the phencyclidine (PCP) binding site. Such compounds have potential as ligands for understanding and possibly the treatment of several neurodegenerative disorders and other glutamate-dependent disorders. We examined the toxic effects of the compounds as compared with memantine, an NMDA receptor antagonist that is FDA approved for treatment of Alzheimer’s disease, by testing these compounds on two cell lines: MDCK (to mimic blood brain barrier) and N2a (a neuronal cell line). The compounds showed toxicity profiles similar to those of memantine *i.e.*, dose dependence above 100 μM and IC_50_ values above 150 μM for each cell line. It is known that the serum level of memantine under therapeutic conditions in patients is about 1 µM, indicting these compounds could have acceptable therapeutic indexes. 2-Phenyl-*N*-(2-(piperidin-1-yl) ethyl)bicyclo[2.2.1]heptan-2-amine (**5a**) was found to possess acceptable toxicity profiles in both cell lines. Interestingly, this was the compound identified as a good lead in our previous studies based on binding and anticonvulsant (MES) activity studies. It has thus emerged as an excellent lead compound for further studies.

## 1. Introduction

**Figure 1:**
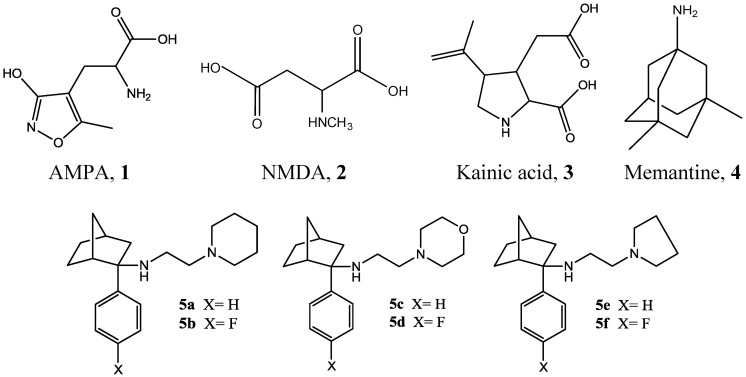
Structures of AMPA, NMDA, kainic acid, memantine and designed novel NMDA receptor antagonists **5a**–**f**.

Glutamate is one of the principal excitatory neurotransmitters in the mammalian central nervous system (CNS). A major function of glutamate is the control of ion flow at excitatory synapses. Three ionotropic receptors for glutamate have been identified based on ligand selectivity: 2-amino-3-(3-hydroxy-5-methyl-isoxazol-4-yl)propanoic acid (AMPA), *N*-methyl-D-aspartatic acid (NMDA), and kainic acid (Figure 1). NMDA receptors are fast-acting, ligand-gated cation channels with a high permeability for Ca^2+^ that are activated by the binding of both L-glutamate and the co-agonist, glycine [1]. NMDA receptors are heteromeric receptors, composed of GluN1 and GluN2 (formerly NR1 and NR2) subunits. Both GluN1 and GluN2 subunits are usually required to create a functional receptor, which usually contains two GluN1 and two GluN2 subunits [2,3].

The NMDA receptor has been implicated in the pathophysiology of a variety of neurological and neuropsychiatric diseases including Alzheimer’s disease (AD) [4], epilepsy, chronic pain syndrome, schizophrenia, Parkinson’s disease, Huntington’s disease [5,6], major depression, addiction, and anxiety [7]. It has also been implicated in central nervous system tumors as observed in neurofibromatosis type 1 (NF1) [8]. Excessive glutamate and subsequent over-stimulation of NMDA receptors leading to excessive Ca^2+^ influx has been implicated in the pathophysiology of these disease states [9,10]. Several preclinical paradigms have found that non-competitive NMDA antagonism can effectively reduce NMDA mediated neurotoxicity [11].

A major limitation for potential therapeutic use of NMDA receptor antagonists is the essential role of the receptor in neuro-physiology. While blockade of excessive NMDA receptor activity is desirable, it must be achieved without complete amelioration of normal glutamate level and attendant functions. As a result, many antagonists have failed in clinical trials [11]. Utilization of uncompetitive antagonists has been proposed as an attractive alternative, as this mechanism requires initial activation of the channel for inhibition to occur, possibly leading to a higher likelihood of channel blockade in the presence of excessive levels of glutamate and a lower likelihood of antagonism with normal physiological levels of glutamate [12]. This approach has had some success as it led to the FDA approved drug for AD, memantine (1-amino-3,5-dimethyladamantine, Namenda^®^, Figure 1) though efficacy and side effects profiles are areas needing improvement.

Our group is involved in the design and syntheses of NMDA receptor antagonists as probes and possible therapies for neurodegenerative diseases with emphasis on AD [13,14,15,16]. We recently reported syntheses of several novel norcamphor (bicycloheptane)-based compounds which were designed as NMDA receptor un-competitive antagonists at the phencyclidine (PCP) biding site [17]. These studies generated six novel target compounds (Figure 1). Both *in vitro* binding and *in vivo* activities were evaluated. The aim of the current study was to examine toxicities of these compounds using cell culture techniques. We wanted to compare *in vitro* toxicities of these compounds with that of memantine and thus determine whether further pursuit of these compounds is warranted, and if so, which one(s). For these studies, we chose two cell lines: epithelial Madin Darby Canine Kidney (MDCK) to mimic blood brain barrier (BBB) and N2a, a neuronal cell line, to assess neurotoxicity [18,19].

## 2. Results and Discussion

Target compounds **5a**–**f** were synthesized as described in our previous publication [17]. Briefly, an appropriately substituted bromobenzene was reacted with magnesium to give a Grignard reagent which was reacted with commercially available norcamphor to give the desired alcohol. The alcohol was then converted to the amine by forming the azide and then reducing it. The amine was then reacted with the alkyl halide to give the target compound. Structures were confirmed using melting point, NMR, and MS techniques. The target compounds are: 2-phenyl-*N*-(2-(piperidin-1-yl) ethyl)bicyclo[2.2.1]heptan-2-amine (**5a**), 2-(4-fluorophenyl-*N*-(2-(piperidin-1-yl) ethyl)bicyclo[2.2.1]heptan-2-amine (**5b**), *N*-(2-morpholinoethyl)-2-phenylbicyclo [2.2.1]heptan-2-amine (**5c**), 2-(4-fluorophenyl)-*N*-(2-morpholino-ethyl)bicyclo [2.2.1]heptan-2-amine (**5d**), 2-phenyl-*N*-(2-(pyrrolidin-1-yl) ethyl) bicyclo[2.2.1]heptan-2-amine (**5e**), and 2-(4-fluorophenyl-*N*-(2-(pyrrolidin-1-yl) ethyl) bicyclo[2.2.1]heptan-2-amine (**5f**).

### 2.1. Toxicities of Novel NMDAR Antagonists and Memantine on MDCK Cells

**Figure 2:**
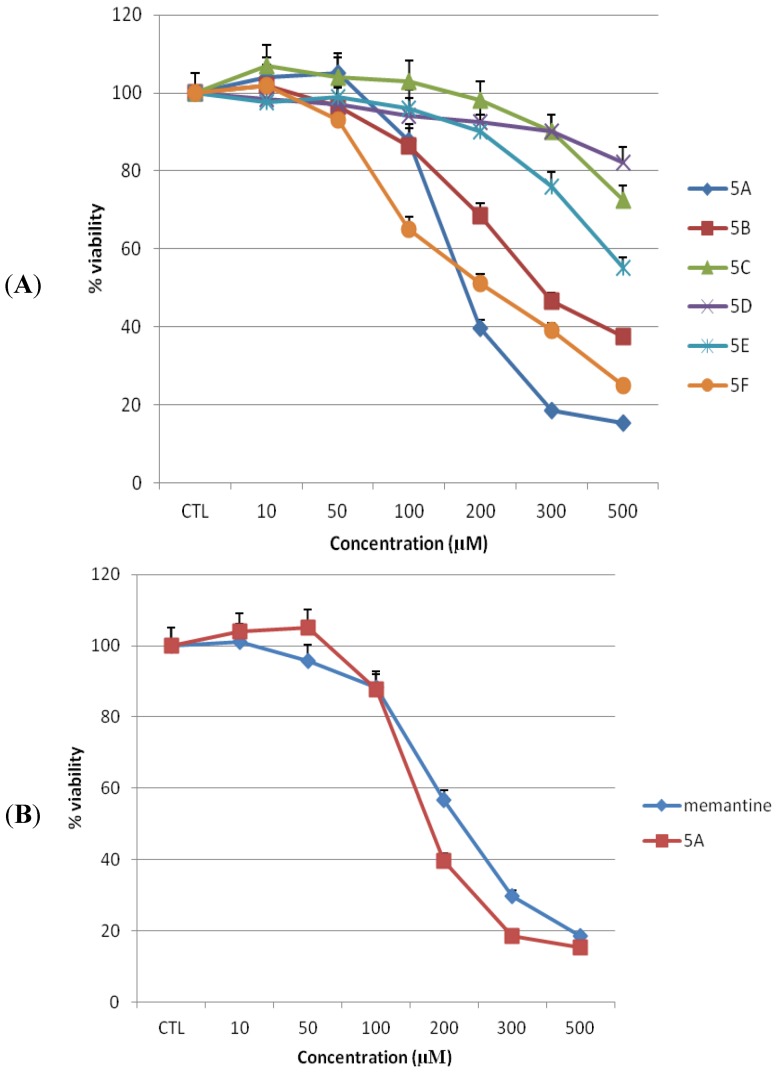
Toxicity of novel NMDA receptor antagonists and memantine on MDCK cells. Toxicity (as the percentage of untreated control) was measured by MTT assay after 24 h of treatment (n = 3). (**A**) Novel NMDA receptor antagonists, **5a**–**f**. (**B**) Lead compound **5a** and memantine. All data represent mean ± S.E.M.

**Figure 3:**
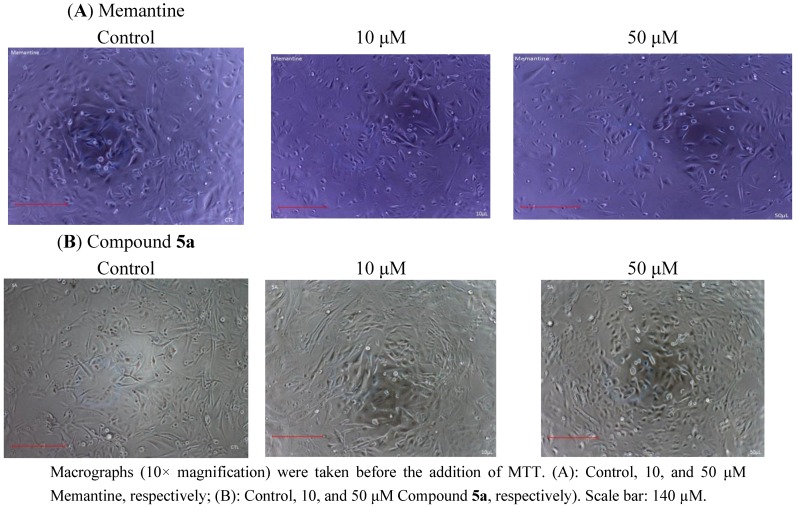
Macrographs using phase contrast microscopy of single point screen at 10 and 50 μM of test compound on MDCK cells.

MDCK cells were used to simulate toxicities of the novel NMDA receptor uncompetitive antagonists **5a**–**f** on BBB *in vitro* [18,19]. We treated cells with 0–500 µM concentrations of test compound or memantine for 24 h. Cell viability was assessed by the capacity of cells to reduce the MTT dye (Figure 2A,B). Treatment with memantine or any of the test compounds resulted in a concentration dependent decline in numbers of viable cells at higher than 100 µM concentration. Our previous studies have indicated that compound **5a** exhibits the highest affinity for NMDA receptor and greatest degree of protection from MES (maximal electroshock)-induced neural damage in rodents [17]. It is known, that under therapeutic conditions in patients, the serum level of memantine is about 1 µM [20]. Since the binding potencies of some of the target compounds are in same range (micromolar) as that of memantine, the 1 µM concentration was used as reference in estimating possible therapeutic indexes. In the present experiments, MDCK cells treated with memantine or compound **5a** at 10 and 50 µM showed no significant reduction in cell viability compared to untreated cells (Figure 2C and Figure 3). The mean percentage of viable cells was 101 ± 0.24 (*p* > 0.05), 95.6 ± 0.25 (*p* > 0.05) for memantine and 104 ± 0.11 (*p* > 0.05), 105 ± 0.06 (*p* > 0.05) for compound **5a** at 10 and 50 µM, respectively, as compared to untreated cells.

### 2.2. Toxicity of Novel NMDAR Antagonists and Memantine on N2a Cells

**Figure 4:**
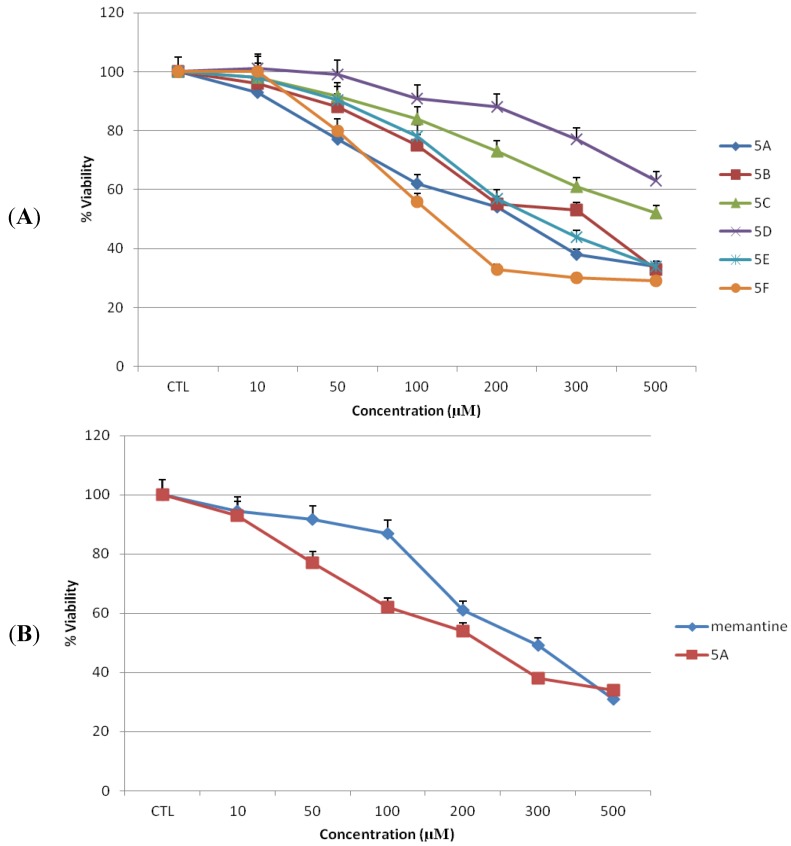
Toxicity of novel NMDA receptor antagonists and memantine on N2a cells. Toxicity (as percentage of untreated control) was measured by MTT assay after 24 h of treatment (n = 3). (**A**) Novel NMDA receptor antagonists **5a**–**f**. (**B**) Lead compound **5a** and memantine. All data represent mean ± S.E.M.

**Figure 5:**
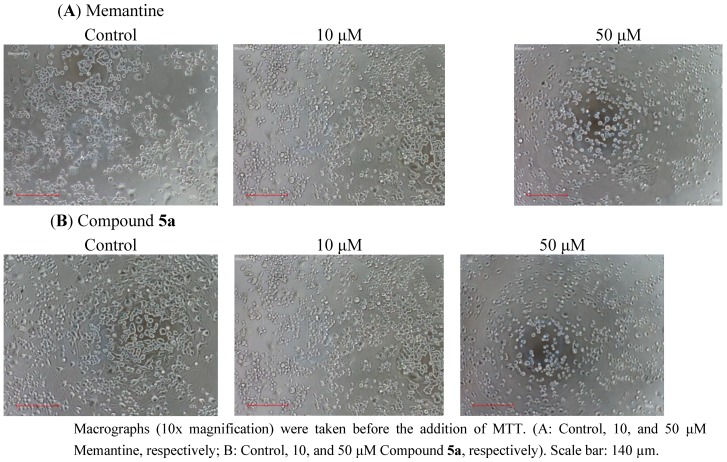
Macrographs using phase contrast microscopy of single point screen at 10 and 50 μM of test compound on N2a cells.

To examine neuronal cell toxicity, N2a cells were exposed to each of compounds **5a**–**f** or memantine for 24 h at 0–500 µM concentrations, and cell viability was measured by MTT assay. In the present experiments, increasing the concentration of antagonists increased the toxicity of both test compounds and memantine in dose dependent manner (Figure 4A,B). N2a cells treated with memantine or compound **5a** at 10, and 50 µM showed no significant reduction in cell viability compared to untreated cells (Figure 4). No significant reduction in cell concentration was observed using phase contrast microscopy (Figure 5). The mean percentage of viable cells was 94.6 ± 0.29 (*p* > 0.05), 91.6 ± 0.26 (*p* > 0.05) for memantine and 93 ± 0.35 (*p* > 0.05), 77 ± 0.35 (*p* > 0.05) for compound **5a** at 10 and 50 µM, respectively, as compared to untreated cells.

Finally, we determined the IC_50_ (concentration yielding 50% inhibition of mitochondrial enzyme activity) values for memantine and novel compounds on both cell lines. The results are listed in Table 1. The IC_50_ values of compound **5a** and memantine on MDCK cells were 155 and 197 µM, respectively, while the values on N2a cells were 154 and 219 µM, respectively. These data indicate that cell toxicity of compound **5a** was comparable to that of memantine. 

**Table 1:** The IC_50_ for novel NMDA receptor antagonists, and memantine on MDCK and N2a cells.

Cell line	Compound	MTT test (IC_50_, µM) ^1)^
**MDCK**	**memantine**	197
	**5a**	155
	**5b**	201
	**5c**	424
	**5d**	>1000
	**5e**	313
	**5f**	305
**N2a**	**memantine**	219
	**5a**	154
	**5b**	291
	**5c**	253
	**5d**	>1000
	**5e**	165
	**5f**	71

^1**)**^ Values are the mean (n = 3) of concentration yielding 50% inhibition of mitochondrial enzyme activity.

## 3. Experimental

### 3.1. Cell Culture

Mouse Neuro-2a (CCL-131, ATCC, Manassas, VA, USA) and MDCK (CCL-34, ATCC) were used in this study. The cells were routinely propagated using Dulbecco’s Modification of Eagle’s Medium (DMEM, ATCC) for N2a and Eagle’s Minimum Essential Medium (EMEM, ATCC) for MDCK cells, supplemented with 10% fetal bovine serum (FBS, ATCC) and 1% penicillin/ streptomycin (Gibco, Grand Island, NY, USA) in 100 cm^2^ Petri-dishes (Corning, Tewksbury, MA, USA) at 37 °C in 5% CO_2_. For neuronal differentiation, N2a cells were cultivated in DMEM supplemented with 2% FBS in the presence of 20 µM retinoic acid (RA, Sigma, St. Louis, MO, USA).

### 3.2. Treatment

All six compounds **5a**–**f** were obtained as salts. Stock solutions (1 mM) were prepared in double distilled water. The treatment concentrations for the compounds and for memantine (Sigma) were 10, 50, 100, 200, 300, and 500 μM. Cells were allowed to attach overnight prior to treatment. H_2_O_2_ served as positive control. Working concentrations were prepared immediately prior to treatment by dilution with medium.

### 3.3. MTT Assay

The MTT (3-(4,5-dimethylthiazole-2-yl)-2,5-diphenyltetrazolium bromide) assay is a well-documented and widely-used cell viability assay. MTT is incorporated into the cell by endocytosis and is reduced by mitochondrial enzymes in living cells to a blue colored formazan precipitate. The absorbance of dissolved formazan in the visible region correlates with the number of intact alive cells [21]. Cells were plated at a concentration of 13,000 to 20,000 cells/well on a 96-well round-bottom plate (Fisher, Pittsburgh, PA, USA). The MTT assay (ATCC, 30-1010K) was performed according to the manufacturer’s instructions. The following determinants were optimized for each cell line: plating cell concentration; incubation time with MTT reagent, and incubation time with detergent reagent. Absorbance was recorded at 570 nm by a microtiter plate reader (VICTOR, PerkinElmer, Waltham, MA, USA). The number of surviving cells is directly proportional to the level of the formazan product generated. Each experiment was repeated on three separate occasions and results are presented as the mean absorbance ± S.E.M.

### 3.4. Phase Contrast Microscopy

The micrographs were taking under a Nikon TS100 inverted microscope using Nikon DS-L2 color camera and Nis-Elements D imaging software. Cells were treated according to experimental design and images were taken before the addition of MTT. A 10X objective was used to analyze the cell concentrations.

### 3.5. Statistical Analysis

The Student’s *t* test was applied to evaluate the significance of differences among each group. A *p* value of 0.05 or less was considered to be significant. Error bars in the graphs are expressed as S.E.M. with experiments performed in triplicate. IC_50_ values were calculated according to the SoftMax Pro GxP analytical software. Standard Curves were plotted using the OD Mean Value (Y) versus Concentration (X) and applying a 4-Parameter fit with a variable weight source curve fit option of 1/Y^2. Results are presented as mean ± S.E.M.

## 4. Conclusions

In the present study we investigated the cytotoxicities of six novel, uncompetitive, NMDA receptor antagonists. These compounds are potential probes and therapies for diseases which can be treated through NMDA receptor antagonism including but not limited to neurodegenerative disorders, depression and cancer. To evaluate *in vitro* toxicities of the novel synthesized compounds, we utilized MDCK (to mimic blood brain barrier and N2a (neural) cell lines. Compounds were evaluated at concentrations of 0–500 μM. Viability was determined by the MTT colorimetric assay, based on the ability of metabolically active cells to convert the pale yellow MTT to a blue formazan product, which is quantifiable spectrophotometrically. IC_50_ values for the compounds were determined. All novel compounds showed toxicity profiles on MDCK and N2a cells similar to those of memantine. When current results are combined with our previous *in vivo* and binding studies, compound **5a** has emerged to be an excellent lead worthy of further pursuit.
